# Sense of coherence and associated factors among university students in China: cross-sectional evidence

**DOI:** 10.1186/s12889-016-3003-3

**Published:** 2016-04-16

**Authors:** Janet Junqing Chu, Mobarak Hossain Khan, Heiko J. Jahn, Alexander Kraemer

**Affiliations:** Department of Public Health Medicine, School of Public Health, Bielefeld University, Universitaetsstr. 25, 33615 Bielefeld, Germany

**Keywords:** Health, Salutogenic, Sense of Coherence, Social support, Stress, University students

## Abstract

**Background:**

Sense of Coherence (SOC) is considered as a health-promoting resource; it is mainly developed before the age of 30. The multiple demands university students face, such as study-related stress and financial difficulty, could challenge their SOC development. This study aimed to: 1) investigate the association between SOC, socio-demographic and lifestyle-related characteristics; 2) assess the effect of perceived stress on SOC controlling for other variables among the Chinese university students. Analyses were done to derive a better view on possible strategies to strengthen students’ SOC and with that to promote their health.

**Methods:**

The data used were from a Chinese university student health survey (*N =* 1,853). Logistic regression analyses were used to explore the effects of varied socio-demographic, lifestyle-related variables on students’ level of SOC, as well as the association between perceived stress and SOC controlling for other variables in the analysis.

**Results:**

Both social support (OR = 2.56 [1.87–3.50]) and better performance compared with peers (OR = 1.64 [1.15–2.34]) were associated with a stronger SOC. Not feeling isolated at university (OR = 1.60 [1.04–2.47]) and satisfaction with the political situation (OR = 2.05 [1.57–2.67]) were also associated with a stronger SOC. This counts also for high health awareness (OR = 1.40 [1.05–1.87]) and nutrition importance (OR = 1.67 [1.04–2.69]). Perceived stress (OR = 0.81 [0.79–0.83]) was negatively associated with a strong SOC when controlling for socio-demographic and lifestyle-related variables.

**Conclusion:**

We suggest integrating stress coping, emotion management training programmes as well as measures promoting social integration for students and teachers at campus, promoting healthy behaviours, and creating a supportive learning environment as strategies for enhancing the SOC level of university students in China.

## Background

### Salutogenic model, sense of coherence, health and university students

Based on the Salutogenic model an individual’s health is determined by the interplay of environmental threats (stimuli), generalized resistance resources (GRRs) at one’s disposal, and the strength of one’s sense of coherence (SOC) [1–3]. SOC has been defined as a construct at the centre of a human information-processing system aimed to resolve conflicts and endure the inevitable stress of human life [1, 4]. This construct is a global orientation to view the world and the individual environment as comprehensible, manageable and meaningful; therefore, SOC reflects three components: comprehensibility, manageability and meaningfulness [1]. A high SOC protects people from stress by the way they perceive life events as challenges not threats (sense of meaningfulness), occurring for a reason not inexplicable (sense of comprehensibility) and that, even if not under their own personal control, they can be handled by some other resource at their disposal instead of feeling overwhelmed and helpless (sense of manageability) [1, 5]. The value of the salutogenic model, the link between SOC and health has been established by research. Studies in Europe and the US that involved large representative samples showed the correlation of SOC with measures of somatic and psychological health [6–9]. The SOC is considered as a health-promoting resource that supports the development of a positive state of mental health [8]. Research shows that mental health promotion is most effective when it takes place early in a person’s life, school-based interventions with a whole setting approach that embrace a more positive view of health can promote health and positive development of youth [10]. The SOC is tested and reinforced mainly in childhood and early adulthood, the years before the age of 30 are the most important period regarding the development of SOC [1, 11]. University students belong to this age group. After coming from a usually familiar environment to the university, an environment with many new demands, students face numerous obstacles and developmental issues such as difficulties of managing academic challenges, deepening relationships with others, expanding social horizon, and choosing and planning a career [12]. Particularly academic tasks and demands at the university have been considered as a normative source of stress that could challenge the SOC of the students. In a longitudinal study among university students in Israel, Carmel & Bernstein [13] found that the SOC level was negatively associated with work load. The authors concluded that the SOC level systematically decreased over time when the work load for the students increased. The university environment has also been reported as a context of socialisation, where people interact with one another and gain practical experiences to increase the ability of drawing the best conclusions in any situations [14]. The process of SOC development has been understood as an internalization of external resources, which were once at the individual’s disposal and ultimately could reduce the present need for other resources [15, 16]. Based on Antonovsky [17] during the development of SOC, each new experience of successful stress management reinforces the SOC of the individual, and offers him/her better coping resources to be available in the future tension events. Our knowledge about how stress perception influences SOC among Chinese higher education students, who undergo relatively high level of perceived stress, remains incomplete. Additionally, although China houses more than one-fifth of the world’s university/college students [18], most of the studies in the last decades focusing on SOC in adolescents and young people were conducted outside China, particularly in Scandinavian countries (44 %) and Israel (21 %) [19]. Therefore, China represents scarcity of information. Furthermore, the present study differs from most of the previous studies, which considered the SOC as a determinant of health. Briefly, as an important attempt to fulfil the abovementioned gaps, this research intended to identify the factors associated with SOC among the university students in China with emphasis on the impact of the perceived stress on the development of SOC. The specific aim was to: 1) investigate the association between SOC, socio-demographic and lifestyle-related characteristics without adjusting for perceived stress—model 1; 2) assess the effect of perceived stress on SOC controlling for other variables—model 2.

### Conceptual framework

**Fig. 1:**
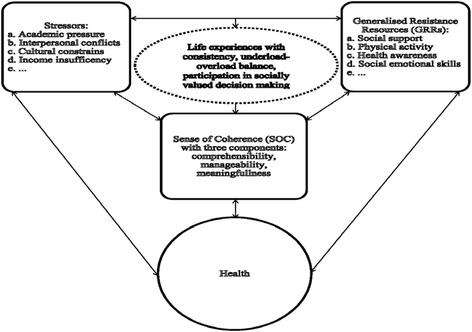
Relation between Stressors, Generalized Resistance Resources and Sense of Coherence (adopted Antonovsky’s Salutogenic model of health [Antonovsky, 1979, p. 184–185])

According to the Salutogenic model, the movement towards the optimal end of the health continuum requires a strong SOC, a good health status in turn can facilitate the acquisition of GRRs, therefore strengthens one’s SOC [17]. Antonovsky stated that the effects of stressors and the GRRs at one’s disposal together shape one’s life experiences [1]. GRRs create life experiences, characterised by consistency, underload-overload balance, and participation in socially valued decision-making, give rise to a strong SOC. The absence of some GRRs, such as money or social ties, can become a stressor. Stressor can also be defined as life experiences characterised by inconsistency, under-, or overload, and exclusion from participation in decision-making [1, 17, 20]. We recapped Antonovsky’s Salutogenic model of health ([1], p. 184–185) in the academic context as Fig. 1. The actual associations of GRRs and stress on SOC may be explained in two directions. In the development of SOC, an individual’s perception of available GRRs intensifies one’s SOC. In turn, a strong SOC enables an individual to mobilize whatever GRRs are at his or her disposal to deal with stressors [1]. Similarly, continual exposure to stressors weakens one’s SOC. On the other hand, individuals with a strong SOC avoid stressors more easily, whereas those with a weak SOC are more likely to interpret stressors as threatening [1]. We are aware that the analysis presented in this paper is not able to test the reciprocal relationship between the factors presented in Fig. 1. Our analysis is driven by the theoretical assumption that a strong SOC is negatively associated with stressors and positively associated with perceived GRRs. It was hypothesised that higher level of perceived stress is associated with lower level of SOC; while GRRs such as social support, good relation with peers, and preventive health orientation such as physical activity and health awareness is positively associated with a strong SOC. Our study findings could provide insight into factors that may refine, reinforce, or modify students’ SOC, therefore assist developing programmes to strengthen the students’ health at Chinese universities.

## Methods

### Study location and questionnaire

The data were obtained from a survey administered in 2010–2011 at two Chinese universities—Sun Yat-sen University (SYSU) in Guangzhou and Peking University (PKU) in Beijing. The employed self-administered questionnaire was an adjusted version of the standardized questionnaire, which was previously used for the Cross National Student Health Study (CNSHS) conducted in seven European countries in 1998–2005. At both universities, roughly one-third of the student sample was from the medical sciences, one-third from allied health sciences (dentistry, nursing, public health and pharmacy), and the rest from the natural sciences and economics with approximately 50 % from each university. The response rate was above 90 % at both universities. Altogether 1,853 students completed the questionnaire. The questionnaire was initially developed in English, in our survey the Chinese version was used. Prior to the survey, the standardized questionnaire was slightly modified to adapt it to the Chinese situation (such as, adding an item asking whether the student is an only-child) and then translated by two independent researchers from English into Chinese. Two bilingual doctoral students examined the translated instruments. The two translated instruments were very close in meaning, indicating correct language transference. A pilot test was also conducted to a group of 32 undergraduate students at PKU before finalising the questionnaire. An ethical approval for the study was obtained from the Institutional Review Boards of Peking University. The students were asked to complete the survey questionnaires at the end of lectures in the lecture rooms. They were informed in writing that participation was voluntary and anonymous; they agreed to participate by completing and returning the questionnaire. No incentives were provided for participation in the survey. The research data used are available in our institutional repository PUB—Publications at Bielefeld University: http://doi.org/10.4119/unibi/2901280.

### Variables

The following variables (with categories in parentheses for categorical variables) were selected for analysis:

#### The sense of coherence (SOC)

The SOC was assessed by the “Leipzig Short Scale” (SOC-L9) based on Antonovsky’s 29-items Sense of Coherence Scale (SOC-29) scale [21]. This unidimensional scale consists of nine items based on a 7-point Likert scale. It has been validated in a representative German population sample of *N =* 2,005 aged 18–92 years old. It was found as a reliable, valid and practicable instrument for measuring the SOC [21]. The SOC-L9 provides a single factor solution with high scores reflecting a strong SOC. A previous Chinese study among university students found a Cronbach’s alpha of 0.73 for the SOC-L9 [22]. The total SOC score ranges from 9 to 63, with higher scores indicating higher SOC. For bivariable analysis and multivariable logistic regression, the total SOC-L9 sum score was dichotomized based on a median split yielding students having a strong SOC (>42) and students having a weak SOC (≤ 42), as the dependent variable. In our sample, Cronbach’s alpha of the SOC-L9 was 0.77.

#### Perceived stress

Perceived stress was assessed by Cohen’s 14-item Perceived Stress Scale (PSS-14), which measures the degree to which a respondent appraises situations in his or her life during the previous four weeks as stressful [23]. These 14 items used 5-point Likert scale responses. The total score (called PSS score) was obtained by adding all 14 items. The score ranges from 0 to 56, where higher scores indicated higher levels of stress. The psychometric properties of the PSS-14 have been evaluated in various cultures and countries with reported Cronbach’s alpha of 0.75–0.89 across studies [24]. Cronbach’s alpha of the PSS-14 scale in our sample was 0.79.

#### Socio-demographic variables

Age, gender, birth place (“countryside”/“small city”/“large city”), Only-child status (“yes”/“no”), having religious beliefs (“yes”/“no”), degree of father’s education (“at least college level” [“high”]/“lower than college level” [“low”]), subjective health status (“good”/“poor”), income sufficiency (“sufficient”/“insufficient”), importance of good grade (“important”/“unimportant”), performance compared with peers (“better”/“the same”/“worse”), having a partner (“yes”/“no”), satisfaction with social support (“satisfied”/“dissatisfied”), relation with parents (“good”/“poor”), relation with fellow students (“good”/“poor”), relation with friends (“good”/“poor”), isolation at university (“yes”/“no”), and satisfaction concerning political situation (“satisfied”/“dissatisfied”).

#### Lifestyle-related variables

Physical activity frequency (“less than once a week”/“once to twice a week”/“at least three times a week”), degree of health awareness (“high”/“low”), importance of nutrition (“important”/“unimportant”), BMI (calculated from self-reported weight and height using Metric BMI Formula: BMI = weight in kilograms divided by the square of height in meters), and satisfaction concerning weight (“satisfied”/“dissatisfied”).

### Statistical analysis

Frequencies and means were tabulated for descriptive analysis. Pearson’s Chi-square tests and Mann–Whitney-U-tests were used to analyse the bivariable associations between SOC (strong vs. weak) and socio-demographic, lifestyle-related variables as well as PSS score. Based on our theoretical model, structural equation modeling (SEM) should be more efficient for the multivariable analysis. As most SEM applications rely on normal theory methods—such as maximum likelihood and generalized least squares—when estimating model parameters and testing model goodness of fit [25]. In the case that observed and latent variables are not normally distributed, standard errors and estimates of fit might not be accurate [26]. The method of Weighted Least Squares offers an alternative, asymptotically distribution-free approach, but a minimum sample size of 2,000 was proposed for obtaining satisfactory results [27]. Since the distribution of the SOC scale sum scores in our subjects was negatively skewed, and our sample was smaller than 2,000, we used SOC scale sum score median as cut-off value for multivariable logistic regression analyses. Binary logistic regression analysis was used by previous studies to assess the association between a strong SOC and relevant variables among university students and in general population [28, 29]. In order to estimate the impact of perceived stress on SOC, we built two multivariable regression models to analyse the adjusted associations of independent variables under investigation with a strong SOC (dependent variable), one model (model 1) excluded perceived stress, and another model (model 2) included perceived stress as an independent variable in the regression. The analysis was performed with IBM SPSS statistics 21. For all tests, the significance level was set at 0.05.

## Results

Sample description is presented in Table 1. There were slightly more males than females (52.1 % vs. 47.9 %) in the sample, the average age of the subjects was 20.8 (± 2.2) years old. Around 90 % of the students rated their health awareness as high, their subjective health, and their relations with parents, friends, as well as fellow students as good. More than 80 % of the students reported sufficient income. The majority (73 %) of the students were satisfied with the social support they received. Only-children accounted for 60 % of the sample. Around 30 % of the students performed physical exercise less than once a week. More than half of the students (56.1 %) were satisfied with the political situation in China. Roughly 17 % of the students felt isolation at the university.

**Table 1:** Sample description

Variables	Categories	Number (%)
Gender	Male	948 (52.1)
	Female	873 (47.9)
Father’s education	High	810 (45.8)
	Low	958 (54.2)
Having religious beliefs	No	1521 (83.7)
	Yes	297 (16.3)
Income sufficiency	Sufficient	1466 (81.2)
	Insufficient	339 (18.8)
Only-child	Yes	1092 (60.0)
	No	727 (40.0)
Birth place	Countryside	596 (32.8)
	Small city	623 (34.3)
	Large city^a^	598 (32.9)
Subjective health	Good	1648 (89.2)
	Poor	200 (10.8)
Health awareness	High	1232 (67.2)
	Low	601 (32.8)
Nutrition importance	Importance	1616 (89.3)
	Unimportance	193 (10.7)
Physical activity	< 1 a week	510 (28.5)
	1–2 a week	872 (48.8)
	≥ 3 a week	406 (22.7)
Weight satisfaction	Satisfied	860 (47.4)
	Dissatisfied	955 (52.6)
Good grade importance	Important	1686 (92.8)
	Unimportant	130 (7.2)
Performance compared with peers	Better	640 (35.3)
	The same	691 (38.1)
	Worse	482 (26.6)
Having a partner	Yes	567 (31.4)
	No	1240 (68.6)
Social support	Satisfied	1336 (73.0)
	Dissatisfied	494 (27.0)
Relation with parents	Good	1652 (89.5)
	Poor	194 (10.5)
Relation with fellow students	Good	1628 (88.1)
	Poor	219 (11.9)
Relation with friends	Good	1676 (90.6)
	Poor	174 (9.4)
Isolation at university	No	1531 (83.1)
	Yes	312 (16.9)
Political situation	Satisfied	1031 (56.1)
	Dissatisfied	808 (43.9)
Sense of coherence scale score	Weak (≤ 42)	953 (52.6)
	Strong (> 42)	859 (47.4)
	Mean (SD)	
Age	20.8 (2.2)	
BMI	20.5 (2.7)	
Perceived stress scale score	24.7 (7.7)	

^a^Cities higher than county level

The results of bivariable analysis between SOC and variables under investigation are presented in Table 2. Income sufficiency and social support were both positively associated with SOC. Students who had high health awareness, had more physical activity and were satisfied with their weight reported stronger SOC. Self-perceived better study performance compared to peers, a good relation with fellow students, not feeling isolated at university, and satisfaction concerning political situation were also associated with a stronger SOC. Neither gender nor BMI was significantly associated with SOC while both age and perceived stress scores were negatively associated with SOC.

**Table 2:** Bivariable analysis: associations of relevant variables with a strong sense of coherence (SOC) (SOC scale score median > 42)

Variables	Categories	SOC scale score median	*p*-value^a^
Gender	Male	42	
	Female	42	0.589
Father’s education	High	43	
	Low	41	0.079
Having religious beliefs	No	42	
	Yes	42	0.205
Income sufficiency	Sufficient	43	
	Insufficient	40	< 0.001
Only-child	Yes	42	
	No	42	0.035
Birth place	Countryside	42	
	Small city	42	
	Large city^b^	43	0.482
Subjective health	Good	43	
	Poor	37	< 0.001
Health awareness	High	43	
	Low	39	< 0.001
Nutrition importance	Important	43	
	Unimportant	37	< 0.001
Physical activity	< 1 a week	40	
	1–2 a week	42	
	≥ 3 a week	44	< 0.001
Weight satisfaction	Satisfied	43	
	Dissatisfied	41	0.015
Good grade importance	Important	42	
	Unimportant	40	0.282
Performance compared with peers	Better	44	
	The same	43	
	Worse	38	< 0.001
Having a partner	Yes	43	
	No	42	0.250
Social support	Satisfied	44	
	Dissatisfied	37	< 0.001
Relation with parents	Good	43	
	Poor	37	< 0.001
Relation with fellow students	Good	43	
	Poor	37	< 0.001
Relation with friends	Good	43	
	Poor	36	< 0.001
Isolation at university	No	43	
	Yes	37	< 0.001
Political situation	Satisfied	44	
	Dissatisfied	39	< 0.001
Age			0.026
BMI			0.292
Perceived stress scale score			< 0.001

^a^Chi square test for categorical variables and Mann–Whitney-*U*-test for continuous variables

^b^Cities higher than county level

Table 3 presents the summary results (ORs and 95 %-confidence intervals) of two multivariable binary logistic regression models using SOC (strong = 1; weak = 0) as an outcome variable. Regression model 1 included gender, age and all other socio-demographic and lifestyle-related variables that were significantly associated with SOC in bivariable analysis. Regression model 2 included all independent variables of model 1 plus PSS score. In both models, preventive health orientation such as high health awareness, paying more attention to nutrition was associated with a strong SOC. No isolation at the university, satisfaction concerning social support and the political situation were also related to a strong SOC. Better performance compared with peers and good subjective health were positively contributed to a strong SOC. The odds of reporting strong SOC was significantly lower among students with higher stress (OR = 0.81 per PSS score point increase) controlling for socio-demographic and lifestyle-related variables. Out of the nine independent variables that showed significant ORs in model 1, seven remained significant in model 2. Adding the PSS score into the regression was helpful to understand the effect of PSS on SOC: It considerably increased the amount of variation explained by the model in the dependent variable—the SOC: Nagelkerke R square from 0.30 in model 1 increased to 0.53 in model 2 (Table 3).

**Table 3:** Odds Ratios (OR) with 95%-confidence interval (95%-CI) for associations of relevant variables with a strong sense of coherence (SOC)

Variables	Categories	Strong SOC- model 1^a^		Strong SOC- model 2^b^	
		OR	95 %-CI	OR	95 %-CI
Gender	Male	1.08	0.87–1.36	1.05	0.81–1.37
	Female (Ref.^c^)				
Income sufficiency	Sufficient	1.32	0.98–1.78	1.13	0.80–1.60
	Insufficient (Ref.)				
Only-child	Yes	1.01	0.81–1.27	0.96	0.73–1.26
	No (Ref.)				
Subjective health	Good	1.94**	1.30–2.89	1.72*	1.07–2.76
	Poor (Ref.)				
Health awareness	High	1.52**	1.18–1.94	1.40*	1.05–1.87
	Low (Ref.)				
Nutrition importance	Important	2.04**	1.34–3.08	1.67*	1.04–2.69
	Unimportant (Ref.)				
Weight satisfaction	Satisfied				
	Dissatisfied (Ref.)	1.02	0.82–1.28	0.93	0.71–1.21
Physical activity	< 1 a week (Ref.)				
	1–2 a week	1.32*	1.01–1.73	1.14	0.83–1.57
	≥ 3 a week	1.61**	1.17–2.21	1.13	0.77–1.66
Performance compared with peers	Better	2.23***	1.66–3.01	1.64**	1.15–2.34
	The same	1.97***	1.48–2.62	1.62**	1.16–2.26
	Worse (Ref.)				
Social support satisfaction	Satisfied	3.20***	2.44–4.21	2.56***	1.87–3.50
	Dissatisfied (Ref.)				
Relation with parents	Good	1.32	0.86–2.03	1.10	0.68–1.79
	Poor (Ref.)				
Relation with fellow students	Good	1.72*	1.05–2.83	1.17	0.67–2.07
	Poor (Ref.)				
Relation with friends	Good	1.17	0.69–2.03	1.04	0.56–1.91
	Poor (Ref.)				
Isolation at university	No	2.54***	1.76–3.69	1.60*	1.04–2.47
	Yes (Ref.)				
Political situation	Satisfied	2.25***	1.80–2.82	2.05***	1.57–2.67
	Dissatisfied (Ref.)				
Age increase per year		0.99	0.94–1.05	0.85	0.89–1.02
PSS score increase per point		-	-	0.81***	0.79–0.83

Model 1 included variables that were significantly associated with SOC in bivariable analysis (except PSS score); model 2 includes perceived stress scale score as an additional independent variable added to model 1

^a^Nagelkerke R square = 0.30 (df = 18, *N =* 1,639, *p <* 0.001)

^b^Nagelkerke R square = 0.53 (df =19, *N =* 1,587, *p <* 0.001)

^c^Reference category; Significance of Wald test: *p < 0.05; **p < 0.01; ***p < 0.001

## Discussion

The SOC is a health-promoting psychological resource that strengthens one’s capacity in dealing with environmental strain and stressful situations [14]. In order to promote health of university students in China by a salutogenic approach, our study tried to identify factors that influence students’ SOC at Chinese universities. Being consistent with previous studies, we found that the SOC was negatively associated with the level of perceived stress [30, 31], and positively associated with academic performance [32, 33]. Similar to a Finnish study, we found that a strong SOC was stronger associated with social support than with socioeconomic status such as family education background, sufficient income, or with merely the existence of relationship such as having a partner [11]. Our findings that more physical activity, paying more attention to health and nutrition were positively associated with the SOC are consistent with the results from two longitudinal studies conducted among university students in Finland and in a general population in Sweden [34, 35]. Our results that being not isolated at the university was related to a strong SOC are in line with the findings of previous studies, that experiences of rejection or exclusion were negatively associated with SOC [36, 37]. Our results that good relation with fellow students was related to a strong SOC are consistent with the findings of a Japanese longitudinal study, generally interpersonal conflict had a negative effect on mean SOC scores [38]. Being consistent with Antonovsky’s [1, 17] SOC theory and the findings of Hassmen et al [39] we found positive association between the SOC, satisfaction concerning political situation and general subjective health. Our results clearly verify the assumption of our conceptual framework: A strong SOC is negatively associated with stressors and positively associated with perceived GRRs; perceived stress has a strong adverse impact on SOC.

### Stress coping, emotion management and SOC

A cross-cultural study suggests that compared to Japanese and Korean students, Chinese students experienced the highest level of stress [40]. Two studies show that the top academic stressors for western students were all related to examination whereas the major stressors among Chinese students were caused by severe competition with peers and the high parental expectation on performance [41, 42]. Our study also suggests that worse academic performance compared with peers and poor relation with fellow students were related to a weaker SOC. In line with the results of previous longitudinal studies that people who experienced higher levels of stress tended to have lower levels of SOC [30, 37], we found that lower level of perceived stress was associated with a strong SOC, perceived stress had a strong impact on explaining the SOC variability. These findings suggest direction for crisis intervention: Shaping stress responses towards the effective coping style which is associated with a strong SOC, and creating a less competitive but more supportive learning environment at university [43].

Coping includes both the ability to mobilise resources to solve the problem (instrumental), and the ability to regulate emotions in the situation (emotional) [44]. Relevant information about stress, coping strategies, and emotion regulation is prerequisite for effective stress management since the availability of knowledge about how to handle difficult situations makes coping efforts more successful [17]. Intervention studies among university students indicated that emotion-related skills (i.e. the perception, use, understanding, and management of emotion) can be deliberately developed and enhanced through social emotional intelligence training [45–47]. Coping is not exclusive to the individual concerned, but also involves interaction between people and the society around them [48]. It was shown that students who have learnt how to control their own emotional reactions and to understand how others feel can deal better when facing difficulties with parents, peers and administrators, therefore consequently increase their “stress tolerance” and “optimism” in life [49]. Previous studies reported that inadequate social skills, lack of interpersonal communication skills, and criticism by others were among the major personal stressors in Chinese university students [40, 42]. It was also reported that support from peers [30, 50] and teachers [51] was positively associated with the development of the SOC in adolescence. Our results concerning the positive association between good relation with fellow students, social support and the SOC are in line with these findings, and also highlight the major role of a good organizational climate at university for enhancing students’ SOC as suggested by Feldt et al [52]. Adolescents and young people need to grow up in an environment that supports them to develop their individual potential, which also provides them with success models to look up to [53]. Socially and emotionally competent teachers can act as a role model to their students for respectful and appropriate interpersonal communication. They may also play an important role in coaching students through conflict situations and help establishing and implementing behavioural guidelines to promote cooperation and intrinsic motivation [49]. It was shown that social emotional intelligence training was an effective approach for promoting teachers’ emotion-related skills and application of these skills in creating a supportive learning environment [49]. While we found that being not isolated at the university is related to a strong SOC, some researchers reported that teacher support and inclusive learning environment were associated with lower levels of stress in adolescents [54]. Therefore, we advocate integrating training programmes regarding stress coping strategy and emotion management for students and faculty into college curriculum.

### Social support, GRRs and SOC

There is some overlap between the concepts of social support, social cohesion and GRRs in literature. Various researchers have defined social cohesion and social support differently, with some definitions much broader than the others [55]. As one of the most widely cited accounts in recent by Jenson ([56], p.15–17) social cohesion includes five dimensions: 1) Belonging (vs. isolation, refers to shared values and identity), 2) inclusion (vs. exclusion, looks at the equality of opportunity), 3) participation (vs. non-involvement, focuses on political participation), 4) recognition (vs. rejection, concerns the respect for diversity), 5) legitimacy (vs. illegitimacy, refers to major political and social institutions act as mediators in conflicts). Social support generally refers to resources supplied to individuals in need by their social network, it can be categorized into four types: 1) emotional support, refers to affect, esteem and concern, 2) instrumental support, reflects the availability of aid in labor, money and time, 3) appraisal support, means feedback and affirmation, and 4) informational support, refers to information, knowledge, and advice that are embedded in social networks [57]. Analytically, social support focuses primarily on the individual and group levels, like the networks maintained by individuals and the personal benefits that flow from them. Social cohesion, on the other hand, requires people’s participation, cooperation and mutual help, is more concerned with the general condition of society [55]. In our study social support was measured as the individual’s perception of the degree to which interpersonal relationships can provide in crisis situations. The GRRs include physical, biological, economic, and psychosocial sources, which can also be divided into internal (personal qualities) and external sources. Some examples of internal personal qualities are e.g. physical strength, knowledge-intelligence, and/or social emotional skills. Similarly examples of external factors are ability to provide social support, opportunities of education, or economic possibilities to satisfy daily needs [15]. Antonovsky [17] also stated that preventive health orientation is an important GRR. Our findings that paying more attention to health and nutrition were associated with a strong SOC support this statement [17]. Knowledge about balanced diet, contraception techniques, and information regarding vaccination station, consulting service at campus should be provided to students routinely as coping resources. We found age is not related to SOC in multivariable analysis, this is in line with the findings of most cross-sectional studies conducted in adolescents [19]. In adult population, some empirical studies found evidence for a continuous process of SOC through life [58, 59]. While other studies pointed out that SOC was relatively stable in the high-SOC group, but not in the low-SOC group [5]. As Morrison & Clift [6] found in a cohort study among adult students that SOC can be improved through peer support learning among students with low entry SOC scores [6].

Compared with the college students in Europe and the USA [60], students in our study reported less frequent physical activity. Meanwhile, our results that physical activity and good relation with fellow students were both positively associated with a strong SOC in model 1, but not in model 2 when PSS score was added into the regression may provide clues about intervention in stress coping and SOC strengthening: Encouraging and facilitating sport events at Chinese campus may have multiple effects on promoting students’ health: through strengthening physical strength, increasing chances for nurturing social support, reducing social isolation as well as interpersonal tension among peers. Based on Antonovsky’s salutogenic concept, consistency, load-balance, and participation in socially-valued decision-making are the elements that provide basis for the three SOC components of comprehensibility, manageability and meaningfulness, respectively [17, 20]. The lack of substantive complexity, disregarding people’s potentials also leads to increasing paralysis of their sense of manageability [17]. In the Chinese context, emphasizing critical thinking, reducing rigidities of the educational system with its reliance on memory-based learning, and deregulating self-organised programs at campus may help strengthening the SOC of the students in particular [61]. Academic freedom and university governance are still the central developmental constraints for Chinese universities [62]. The extent to which people have a voice in what goes around them has significant influence on their level of meaningfulness [17]. Over control from the state on universities may discourage the participation of students in decision-making in their academic as well as social life in China, thus may negatively contribute to the development of a strong SOC within this population. Our finding that dissatisfaction with the political situation in China was related to weaker SOC of university students may give a hint in this regard. On the other hand stressors are more challenges to be welcomed than burden to be escaped in traditional Chinese cultural value, as the well-known philosophy of Mencius said: Whenever heaven invests an individual with great responsibilities, it first tries his/her resolve, exhausts his/her muscles and bones, starves his/her body, leaves him/her destitute, and confounds his/her every endeavor. In this way his/her patience and endurance are developed, and his/her weakness is overcome [63]. According to Antonovsky [53], a complex culture can be a potential stressor but it also offers many possibilities of choice. Thus, for people who are flexible in choosing strategies, cultural constrains can even be SOC promoting. For instance, sharing social risk, pressure and burden of an interdependent society facilitates solidarity of the affected [64]. As Antonovsky suggested, high stressors can be salutary when accompanied by high levels of social supports [1].

### Limitations

Our results must be interpreted in relation to some potential methodological limitations. The first limitation involves cultural adaptation, since we added two additional socio-demographic variables (only-child status and birth place) to the CNSHS questionnaire. This minor modification was necessary to respond the specific Chinese context, e.g. 60 % of our subjects were only-children, as reported elsewhere that only-child status is associated with students’ peer relation and their integration at the university [65]. Nevertheless, the main variables under investigation—SOC and PSS scales—should be culturally comparable since we used the official Chinese version of the two scales which are reliable, valid, and cross culturally applicable [24, 48]. The second limitation concerns the sample, taken only from two universities, was over-represented by the students of medical and health sciences. Therefore, our results may not be transferable to all university students in China. Regarding the third limitation, given the cross-sectional character of our study, the results allow only conclusions about associations and not about causations. Finally, our data are based on self-reported information what may to a certain extent result in socially desired answers (in spite of the anonymous data collection situation) or incorrect information due to recall bias. However, despite these limitations our research has important strength. As to the best of our knowledge, no previous study have investigated in detail the associations of SOC with perceived stress, a wide range of socio-demographic and with lifestyle-related characteristics in a large sample of Chinese university students.

## Conclusion

In conclusion, our study explored the effects of varied socio-demographic, lifestyle-related variables as well as perceived stress on students’ level of SOC in university sitting in China. As reported in this paper, lower level of perceived stress, preventive health orientations, as well as integration at university are all related to a strong SOC. It is hoped that our findings could be useful in providing guidelines for interventions aiming to strengthen students’ SOC, consequently to promote the health of students at Chinese universities.

## Abbreviations

BMI: body mass index
CNSHS: the cross national student health study
GRRs: generalized resistance resources
OR: odds ratio
PKU: Peking University
PSS: perceived stress scale
SEM: structural equation modeling
SOC: sense of coherence
SYSU: Sun Yat-sen University
95 %-CI: 95 %-confidence interval
